# Combining microorganisms in inoculants is agronomically important but industrially challenging: case study of a composite inoculant containing *Bradyrhizobium* and *Azospirillum* for the soybean crop

**DOI:** 10.1186/s13568-021-01230-8

**Published:** 2021-05-22

**Authors:** Marcos Vinicios Conceição Garcia, Marco Antonio Nogueira, Mariangela Hungria

**Affiliations:** 1 Department of Biotechnology, Universidade Estadual de Londrina, C.P. 10011, Londrina, Paraná 86057-970 Brazil; 2 Soil Biotechnology Laboratory, Embrapa Soja, C.P. 231, Londrina, Paraná 86001-970 Brazil

**Keywords:** Biological nitrogen fixation, Plant-growth promoting bacteria, Inoculation, Industrial microbiology

## Abstract

The increasing global perception of the importance of microbial inoculants to promote productivity and sustainability in agriculture prompts the adoption of bio-inputs by the farmers. The utilization of selected elite strains of nitrogen-fixing and other plant-growth promoting microorganisms in single inoculants creates a promising market for composite inoculants. However, combining microorganisms with different physiological and nutritional needs requires biotechnological development. We report the development of a composite inoculant containing *Bradyrhizobium diazoefficiens* and *Azospirillum brasilense* for the soybean crop. Evaluation of use of carbon sources indicates differences between the microbial species, with *Bradyrhizobium* growing better with mannitol and glycerol, and *Azospirillum* with malic acid and maleic acid, allowing the design of a formulation for co-culture. Species also differ in their growth rates, and the best performance of both microorganisms occurred when *Azospirillum* was inoculated on the third day of growth of *Bradyrhizobium*. The composite inoculant developed was evaluated in five field trials performed in Brazil, including areas without and with naturalized populations of *Bradyrhizobium*. The composite inoculant resulted in symbiotic performance comparable to the application of the two microorganisms separately. In comparison to the single inoculation with *Bradyrhizobium*, co-inoculation resulted in average increases of 14.7% in grain yield and 16.4% in total N accumulated in the grains. The performance of the composite inoculant was similar or greater than that of the non-inoculated control receiving a high dose of N-fertilizer, indicating the importance of the development and validation of inoculants carrying multiple beneficial microorganisms.

**Supplementary Information:**

The online version contains supplementary material available at 10.1186/s13568-021-01230-8.

## Introduction

The global search for low-cost agricultural technologies that can help increase food offer under sustainable production models is becoming more relevant (Sá et al. 2017). Microorganisms are major players in this vision of agriculture of the future and, in fact, in the last decade changes in farmers´ perception have already been noticed, reflecting in increased adoption of microbial bio-inputs (Malusá and Vassilevde 2014; de Bruijn 2015; Fukami et al. 2018a; Bellabarba et al. 2019; Santos et al. 2019).

In the coming years, Brazil should consolidate its position as a major player in the production and commercialization of food, in addition to standing out in the production of fibers, biofuels and biomass. Indeed, the country has just become the world's largest soybean producer [*Glycine max* (L.) Merr.] (USDA 2020). The effort in the development of research with microorganisms of agricultural importance has been a constant in the country, and a great example is the adoption of the process of biological N_2_ fixation (BNF) for the economic viability of the soybean crop, making it independent of N-fertilizers. The savings provided by the BNF with the soybean crop in Brazil are estimated today at US $ 14.4 billion per crop. Noticeable is also the environmental contribution, lowering the emission of greenhouse gases and reducing the leaching of nitrate to rivers, water reservoirs, groundwater, and lakes (Hungria et al. 2013a; Hungria and Mendes 2015; Sá et al. 2017; Hungria and Nogueira 2019).

Considering the soybean crop, three major milestones in the recent history of BNF in Brazil can be highlighted. The first is the validation of the reinoculation technology, with an average 8% increase in grain yield by inoculation every year (Hungria and Mendes 2015; Hungria and Nogueira 2019; Hungria et al. 2006, 2007, 2020). The second came from the selection of the first commercial strains of *Azospirillum brasilense* for grasses, with the release of the first commercial product in 2009 (Hungria et al. 2010; Santos et al. 2019, 2021). The third major milestone was the development of the technology of co-inoculation for the soybean in 2013, consisting of the combination of strains of *Bradyrhizobium* spp. highly efficient in fixing nitrogen and strains of *A. brasilense* with high phytohormone production capacity (Hungria et al. 2013b, 2015). In the short period of five years, co-inoculation has been adopted on average in 25% of the 36 million ha cropped with this legume (Santos et al. 2021).

There is no doubt that the combination of microbial inoculants can provide excellent results, justifying their great potential of being employed worldwide (Bashan 1998; Juge et al. 2012; Malusá and Vassilevde 2014; Fukami et al. 2017; Bellabarba et al. 2019; Santos et al. 2019, 2021). Farmers, however, demand more practicality at sowing, and are requesting commercial products that combine various microorganisms. However, it is not easy to combine microorganisms with different nutritional requirements and growth rates. A strategy is presented in this study, where the stages of development and field validation of a composite inoculant containing *Bradyrhizobium* and *Azospirillum* for the soybean crop are presented.

## Material and methods

### Strains used in the study

The study included the strains of *Bradyrhizobium japonicum* SEMIA 5079 (= CNPSo 7, = CPAC 15), *Bradyrhizobium diazoefficiens* SEMIA 5080 (= CNPSo 6, = CPAC 7), and *Azospirillum brasilense* strains Ab-V5 (= CNPSo 2083) e Ab-V6 (= CNPSo 2084), all employed in commercial inoculants in Brazil for legumes and grasses. The strains are deposited in the “Diazotrophic and Plant Growth Promoting Bacteria Culture Collection of Embrapa Soja” (WFCC Collection # 1213, WDCM Collection # 1054).

### Development of the inoculant

#### Preparation of pre-inocula of Bradyrhizobium spp. and A. brasilense

Pre-inocula were prepared to initiate bacterial cultures for the studies of C sources and inoculation time.

Strains SEMIA 5079 and SEMIA 5080 of *Bradyrhizobium* spp. were grown in modified-YM broth (Hungria et al. 2016), and *A. brasilense* strains Ab-V5 and Ab-V6 in modified-RC (*Rojo Congo*) broth (Santos et al. 2020), without Congo red, in 100 mL bottles. The samples were incubated at 28 ℃ ± 2 ℃, for 5 days and 3 days, respectively, on a rotary shaker at 120 rpm. After growth, the cultures were centrifuged for 10 min at 6000 rpm. The supernatant was discarded and the pellet resuspended in 0.85% NaCl saline solution and centrifuged again, under the same conditions. The supernatant formed in the second centrifugation was discarded, re-suspending the pellet in 40 mL of the respective media, in order to adjust the concentration to approximately 10^9^ cells mL^−1^ for *Bradyrhizobium* and 10^8^ cells mL^−1^ for *Azospirillum*. The concentration was adjusted by reading the optical density (O.D.) on a spectrophotometer at 600 nm, according to growth curves previously prepared and available in the laboratory. Concentrations were read at O.D. 0.400 for SEMIA 5080, O.D. 0.600 for SEMIA 5079 (higher production of exopolysaccharides) and O.D. 0.600 for both strains of *Azospirillum*.

### Growth in different carbon sources and elaboration of a basic formulation of coinoculant

In order to evaluate the ability to metabolize different carbon sources, 10 mL pre-inoculum of each strain were added to 100 mL of the appropriate medium, at pH 6.8, containing different C sources (Table 1), in 500 mL flasks. Cultures were grown on a rotary shaker at 120 rpm, at 28 ℃ ± 2 ℃. The number of colony forming units (CFU) present in each medium was determined from aliquots aseptically removed from the cultures at different intervals during growth, by means of the spread-plate technique (O´Hara et al. 2016), in modified-YMA solid medium (Hungria et al. 2016) for *Bradyrhizobium* and RC solid medium (Santos et al. 2020) for *Azospirillum*. In addition, O.D. and the pH of the culture media were also evaluated.

**Table 1:** Composition of the culture media to verify the use of different sources of carbon by *Bradyrhizobium japonicum*, *Bradyrhizobium diazoefficiens* and *Azospirillum brasilense*

Component	Treatments (g L^−1^)								
	T1	T2	T3	T4	T5	T6	T7	T8	T9
K_2_HPO4	0.5	0.5	0.5	0.5	0.5	0.5	0.5	0.5	0.5
MgSO_4_.7H_2_O	0.2	0.2	0.2	0.2	0.2	0.2	0.2	0.2	0.2
NaCl	0.1	0.1	0.1	0.1	0.1	0.1	0.1	0.1	0.1
Yeast extract	0.0	0.5	0.5	0.5	0.5	0.5	0.5	0.5	0.5
Sucrose	0.0	0.0	5.0	0.0	0.0	0.0	0.0	0.0	0.0
Glycerol	0.0	0.0	0.0	5.0	0.0	0.0	0.0	0.0	0.0
Mannitol	0.0	0.0	0.0	0.0	5.0	0.0	0.0	0.0	0.0
Glutamic acid	0.0	0.0	0.0	0.0	0.0	5.0	0.0	0.0	0.0
Malic acid	0.0	0.0	0.0	0.0	0.0	0.0	5.0	0.0	0.0
Citric acid	0.0	0.0	0.0	0.0	0.0	0.0	0.0	5.0	0.0
Maleic acid	0.0	0.0	0.0	0.0	0.0	0.0	0.0	0.0	5.0

From the results obtained, six formulations were developed that allowed the growth of both *Bradyrhizobium* and *Azospirillum*.

### Determination of the sequence of inoculation of Bradyrhizobium and Azospirillum in the formulation of the composite inoculant

*Bradyrhizobium* spp. and *A. brasilense* exhibit different growth rates. It is also known that the concentration of *Azospirillum* strains of Ab-V5 and Ab-V6, when used for co-inoculation of soybean, must be ten times lower than that of *Bradyrhizobium* spp to optimize growth and BNF (Hungria et al. 2013b). In order to achieve these conditions, co-cultivation tests were carried out with the two best formulations identified in the experiments of growth with different C sources, to determine the appropriate times for sequential inoculation of the two species.

The experiment started with the inoculation of 10 mL of pre-inoculum of *Bradyrhizobium* spp. at the concentration of 1 × 10^8^ UFC mL^−1^ in each of four flasks containing of 100 mL culture medium, and this was considered as the initial cultivation, or zero time. Following, 10 mL of the pre-inoculum of *A. brasilense* strains Ab-V5 and Ab-V6 at the concentration of 1 × 10^7^ UFC mL^−1^ were inoculated in the flasks containing *Bradyrhizobium* spp. growing for 2 (T1), 3 (T2) and 4 (T3) days. The control treatment consisted only of *Bradyrhizobium* spp., and received no *A. brasilense*.

The analyzes of cell concentration of the control treatment were performed from the second to the fifth day of cultivation of *Bradyrhizobium* spp., whereas, for the treatments of co-culture T1, T2 and T3, the analyzes were carried out after 24 h of each time of inoculation of *A. brasilense*. Growth assessments were carried out as described in the item of evaluation of growth with different carbon sources.

### Evaluation of the composite inoculant in field trials

#### Treatments

For the field experiments, the co-inoculant evaluated corresponded to the best formulation identified under laboratory conditions and carrying the strain of *B. diazoefficiens* SEMIA 5080 and *A. brasilense* Ab-V6.

The treatments evaluated were: T1—Non-inoculated control without N-fertilizer; T2—Non-inoculated control receiving 200 kg of N ha^−1^ of N-fertilizer (supplied as urea, 50% at sowing, and 50% at flowering); T3—Peat inoculant (treatment required by Brazilian legislation, considered as “gold standard”, e.g. Burton and Curley 1965) containing the strains of *B. japonicum* SEMIA 5079 and *B. diazoefficiens* SEMIA 5080, applied to supply 1.2 × 10^6^ cells seed^−1^; T4—Trademark commercial liquid inoculant containing the strains of *B. japonicum* SEMIA 5079 and *B. diazoefficiens* SEMIA 5080, applied to supply 1.2 × 10^6^ cells seed^−1^; T5—Trademark commercial co-inoculant consisting of individual packs of *Bradyrhizobium* and *Azospirillum*, the first containing the strains of *B. japonicum* SEMIA 5079 and *B. diazoefficiens* SEMIA 5080, applied to provide 1.2 × 10^6^ cells seed^−1^, and the second with *A. brasilense* Ab-V5 and Ab-V6, applied to supply 1.2 × 10^5^ cells seed^−1^; T6—Composite inoculant developed in this study applied to supply 1.2 × 10^6^ cells seed-1 of *B. diazoefficien*s SEMIA 5080 and 1.2 × 10^5^ cells seed^−1^ of *A. brasilense* Ab-V6. An extra-treatment (T7), consisting of in-furrow application of a triple dose of the composite inoculant developed in this study was included in two sites.

### Establishment and conduction of the field trials

Five field trials were conducted in the 2017/2018 crop season, at five different sites; the geographic coordinates, climatic conditions, characteristics and soil classification of each location are presented in Additional file 1: Table S1.

At each site, between 40 and 60 days before the beginning of the experiment, 20 soil sub-samples were obtained, in the layers of 0–20 and 20–40 cm, to evaluate the chemical and soil granulometric properties of the soil.

The chemical analysis of the soils and the reference values were defined according to van Raij et al. (2001) and Silva (2009) and the results obtained are presented in Additional file 1: Tables S2 and S3. The C content in the soil was determined in an elementary analyzer Vario TOC Cube in air-dried and finely ground samples (< 0, 02 mm). This method is considered suitable for soils with high Fe content, due to its greater accuracy and reproducibility (Segnini et al. 2008). The results obtained are available in Additional file 1: Table S3. Regarding the granulometric analysis, the fractions of sand, silt and clay were determined according to Teixeira et al. (2017) and the results are shown in Additional file 1: Table S3.

According to the results obtained in the soil analysis, liming was performed, estimated to reach base saturation of 70% in Londrina, 60% in Lutécia, and 50% in the other sites (Embrapa Soja 2013). Approximately 30 days before sowing, weed control was carried out with the application of 2.5 L ha^−1^ of glyphosate.

Immediately before sowing, fertilization was carried out with 300 kg ha^−1^ of the formulation 00-20-20 (60 kg ha^−1^ of P_2_O_5_ and 60 kg ha^−1^ of K_2_O) for all treatments. In treatment 2 (T2), non-inoculated control receiving N-fertilizer, 100 kg of N ha^−1^ (urea) were applied to the surface of the plot and lightly incorporated.

The population of rhizobia symbionts of soybean in the 0–10 cm soil layer was evaluated in samplings carried out in the same way as for the chemical analysis and soil granulometry, but collected on the sowing day. The population was estimated by the most probable number (MPN) method, with counts on soybean plants of the cultivar BRS 1010IPRO (Hungria et al. 2016). In the same samples, the population of diazotrophic bacteria was also evaluated by MPN method in semi-solid NFb culture medium (Döbereiner et al. 1976). The results obtained are presented in Additional file 1: Table S3.

The soybean cultivar used in the field experiments was the Embrapa BRS 1010IPRO, transgenic, with tolerance to the glyphosate herbicide and with the Intacta RR2 PROTM technology for caterpillar control. The size of the plots, the sowing dates, and the density of plants at each location are presented in Additional file 1: Table S4. Plots and blocks were interspaced by 1 or 2 m corridors to avoid contamination of treatments.

The experimental design of the trials was randomized complete block design (RCBD), with six replications.

In the phenological stages V3–V5 (Fehr and Caviness 1977), foliar application of Co (2.5 g ha^−1^) + Mo (20 g ha^−1^) was performed. No other micronutrients were applied. Approximately in R1 (Fehr and Caviness 1977), the T2 (non-inoculated control with N-fertilizer), received the second dose of N, of 100 kg of N ha^−1^ as urea, side-dressed.

All cultural and phytosanitary treatments were carried out as specified in the technologies for soybean production (Embrapa Soja 2013).

### Plant sampling and analyses

Between 42 and 49 days after sowing (DAS), corresponding to 35 to 36 days after emergence (DAE) (Additional file 1: Table S4), when the plants were in the V5 growth stage (Fehr and Caviness 1977), five plants were collected from each plot. Evaluations at this sampling included nodulation (number and dry weight), shoot dry weight, N content and total N accumulated in the shoot.

In the laboratory, shoots and the roots were separated, washed carefully and allowed to dry in an oven at 50 ℃, until they reached constant dry weight (approximately 72 h). The nodules were removed from the roots, dried again and weighed to obtain nodule dry weight. The N content in shoot (N-Kjeldahl) was determined in a spectrophotometer by the salicylate green method, with reading at 697 nm (Feigl and Anger 1972). The N content multiplied by the biomass of the aerial part of the plants resulted in the N accumulated in shoots.

Grain yield was determined at the physiological maturation, harvesting the central area of each plot dates, ranging from 6.75 to 8 m^2^, as shown in Additional file 1: Table S4. The grains were cleaned, weighed and, after determining the moisture in a grain moisture determiner (Gehaka, model AGRI G800), the mass was corrected to 13% moisture. The N content in the grains was also determined (N-Kjeldahl) according to Feigl and Anger (1972). The N content multiplied by the grain dry weight resulted in the N accumulated in the grains.

### Statistical analysis

The data from the experiments to analyze the use of different C sources and the time of inoculation were subjected to the test of normality and homogeneity of variances. Then, analysis of variance (ANOVA) was performed. When the “F” test was significant at 5%, the treatment meanswere compared by the Tukey test at 5%, using the statistical program R.

The data from each field trial were initially submitted to the test of normality of variables and homogeneity of variances. Then, analysis of variance (ANOVA) was carried out according to a randomized block design using the software Statistica version 7.0. When the “F” test was significant at 5%, the treatment means were compared by the Duncan test, at the 5% level of significance. When the “F” test was not significant at 5%, the significance was verified at 10%, as specified in Brazilian legislation for agronomic efficiency tests for the registration of inoculants (MAPA 2011), to compare the means at the level of 10% of significance. At the end, a joint analysis of the five trials was also carried out, using the same statistical tests.

## Results

### Development of the co-inoculant formulation

In the first stage of the study, as the inoculants produced in Brazil for the soybean crop contain two strains of *Bradyrhizobium*, mostly *B. japonicum* SEMIA 5079 and *B. diazoefficiens* SEMIA 5080, and two strains of *A. brasilense*, Ab-V5 and Ab-V6, the capacity to metabolize C sources was evaluated for each pair of strains. The genera differed in their ability to use C sources. For *Bradyrhizobium*, higher cell concentrations were achieved in the presence of sucrose, mannitol and glycerol (Table 2). For these sources, in the evaluation performed at 34 days, alkalinization of the culture medium formulated with organic acids was detected, varying from pH 8.17 to 9.27, with less variation with mannitol, sucrose and yeast extract (data not shown).

**Table 2:** Cell concentration (UFC mL^−1^) of *Bradyrhizobium* spp. strains SEMIA 5079 and SEMIA 5080 growing with different carbon sources for 34 days

Days	Carbon source								
	Control	Yeast extract	Sucrose	Glycerol	Mannitol	Glutamic acid	Malic acid	Citric acid	Maleic acid
2	1.21 × 10^8^	6.44 × 10^8^	7.66 × 10^8^	7.88 × 10^8^	1.02 × 10^9^	3.22 × 10^8^	9.43 × 10^8^	1.07 × 10^8^	3.55 × 10^8^
3	3.10 × 10^8^	9.10 × 10^8^	1.69 × 10^9^	1.04 × 10^9^	9.29 × 10^8^	1.99 × 10^8^	4.22 × 10^8^	1.66 × 10^8^	8.75 × 10^7^
4	1.01 × 10^8^	3.22 × 10^8^	1.78 × 10^9^	1.41 × 10^9^	1.27 × 10^9^	4.22 × 10^8^	3.55 × 10^8^	1.11 × 10^8^	8.77 × 10^7^
5	1.13 × 10^8^	9.32 × 10^8^	2.44 × 10^9^	1.18 × 10^9^	1.22 × 10^9^	3.88 × 10^8^	4.55 × 10^8^	9.64 × 10^7^	6.66 × 10^7^
6	1.03 × 10^8^	6.88 × 10^8^	1.78 × 10^9^	1.01 × 10^9^	1.08 × 10^9^	5.44 × 10^8^	3.66 × 10^8^	7.33 × 10^7^	5.11 × 10^7^
7	1.95 × 10^8^	4.77 × 10^8^	1.81 × 10^9^	1.08 × 10^9^	9.74 × 10^8^	5.33 × 10^8^	3.33 × 10^8^	7.88 × 10^7^	3.77 × 10^7^
9	3.12 × 10^7^	5.27 × 10^8^	1.71 × 10^9^	1.05 × 10^9^	1.03 × 10^9^	8.09 × 10^8^	3.04 × 10^8^	6.58 × 10^7^	2.89 × 10^7^
10	–^a^	7.10 × 10^8^	1.77 × 10^9^	1.33 × 10^9^	1.08 × 10^9^	1.06 × 10^9^	2.77 × 10^8^	6.22 × 10^7^	2.11 × 10^7^
11	–	8.76 × 10^8^	2.10 × 10^9^	1.53 × 10^9^	1.76 × 10^9^	1.38 × 10^9^	1.55 × 10^8^	5.66 × 10^7^	2.22 × 10^7^
12	–	7.44 × 10^8^	2.01 × 10^9^	1.18 × 10^9^	1.03 × 10^9^	1.17 × 10^9^	4.55 × 10^7^	5.55 × 10^7^	1.99 × 10^7^
13	–	7.77 × 10^8^	3.33 × 10^9^	1.27 × 10^9^	1.03 × 10^9^	1.08 × 10^9^	2.55 × 10^7^	3.32 × 10^7^	1.22 × 10^7^
20	–	4.77 × 10^8^	2.44 × 10^9^	1.29 × 10^8^	8.77 × 10^8^	2.66 × 10^8^	–	9.98 × 10^6^	3.44 × 10^6^
23	–	6.88 × 10^8^	2.04 × 10^9^	1.36 × 10^9^	1.07 × 10^9^	1.20 × 10^8^	–	–	–
25	–	5.55 × 10^8^	1.54 × 10^9^	1.25 × 10^9^	1.18 × 10^9^	6.22 × 10^7^	–	–	–
27	–	6.11 × 10^8^	1.41 × 10^9^	1.14 × 10^9^	1.11 × 10^9^	2.99 × 10^7^	–	–	–
34	–	3.33 × 10^8^	1.26 × 10^9^	5.88 × 10^8^	8.55 × 10^8^	4.44 × 10^6^	–	–	–

^a^Traces indicate absence of growth

For *Azospirillum*, the best growth was obtained with maleic acid and malic acid, but adequate cell concentrations were also obtained with sucrose, yeast extract and mannitol (Table 3). In general, there was alkalinization of the culture medium, varying from pH 8.05 for yeast extract, to 8.79 with maleic acid, and the only exception was glycerol, with lower pH 7.01 (data not shown).

**Table 3:** Cell concentration (UFC mL^−1^) of *Azospirillum brasilense* strains Ab-V5 e Ab-V6 growing with different carbon sources for 34 days

Days	Carbon source								
	Control	Yeast extract	Sucrose	Glycerol	Mannitol	Glutamic acid	Malic acid	Citric acid	Maleic acid
1	3.99 × 10^7^	6.88 × 10^8^	6.99 × 10^8^	8.66 × 10^8^	5.33 × 10^8^	1.63 × 10^9^	6.55 × 10^8^	5.11 × 10^8^	6.44 × 10^8^
2	5.30 × 10^7^	4.55 × 10^8^	4.10 × 10^8^	1.11 × 10^8^	5.88 × 10^8^	1.58 × 10^9^	5.66 × 10^8^	1.48 × 10^8^	6.11 × 10^8^
3	7.88 × 10^7^	4.10 × 10^8^	3.33 × 10^8^	9.98 × 10^7^	4.21 × 10^8^	1.70 × 10^9^	2.77 × 10^8^	1.07 × 10^8^	5.11 × 10^8^
6	2.22 × 10^7^	2.44 × 10^8^	1.18 × 10^8^	6.75 × 10^7^	4.22 × 10^8^	7.66 × 10^8^	1.41 × 10^8^	4.85 × 10^6^	4.33 × 10^8^
7	6.55 × 10^6^	2.99 × 10^8^	1.02 × 10^8^	7.76 × 10^7^	1.65 × 10^8^	9.86 × 10^8^	9.75 × 10^7^	–	4.77 × 10^8^
9	–^a^	4.77 × 10^7^	1.01 × 10^8^	6.66 × 10^6^	3.79 × 10^7^	5.12 × 10^7^	8.21 × 10^7^	–	2.33 × 10^8^
10	–	4.77 × 10^7^	9.39 × 10^7^	–	4.21 × 10^7^	8.55 × 10^7^	5.69 × 10^7^	–	2.99 × 10^8^
13	–	2.77 × 10^7^	9.63 × 10^7^	–	2.77 × 10^7^	4.44 × 10^6^	3.33 × 10^8^	–	1.55 × 10^8^
14	–	3.77 × 10^7^	7.70 × 10^7^	–	1.95 × 10^7^	5.66 × 10^6^	8.43 × 10^7^	–	1.36 × 10^8^
15	–	3.22 × 10^7^	9.98 × 10^7^	–	2.44 × 10^7^	4.44 × 10^6^	1.01 × 10^8^	–	1.65 × 10^8^
18	–	5.00 × 10^7^	1.07 × 10^8^	–	1.44 × 10^7^	–	4.55 × 10^7^	–	1.39 × 10^8^
21	–	2.77 × 10^7^	6.66 × 10^7^	–	1.77 × 10^7^	–	9.21 × 10^7^	–	4.44 × 10^7^
22	–	3.33 × 10^7^	8.44 × 10^7^	–	2.99 × 10^7^	–	1.03 × 10^8^	–	1.04 × 10^8^
23	–	3.11 × 10^7^	9.63 × 10^7^	–	2.66 × 10^7^	–	9.10 × 10^7^	–	1.05 × 10^8^
27	–	2.33 × 10^7^	4.77 × 10^7^	–	1.77 × 10^7^	–	7.22 × 10^7^	–	4.88 × 10^7^
28	–	1.99 × 10^7^	5.88 × 10^7^	–	2.77 × 10^7^	–	3.77 × 10^7^	–	6.66 × 10^7^
30	–	2.22 × 10^7^	4.66 × 10^7^	–	2.66 × 10^7^	–	5.22 × 10^7^	–	3.88 × 10^7^
31	–	3.22 × 10^7^	5.44 × 10^7^	–	1.77 × 10^7^	–	6.66 × 10^7^	–	4.22 × 10^7^
34	–	2.88 × 10^7^	4.55 × 10^7^	–	1.66 × 10^7^	–	6.11 × 10^7^	–	6.55 × 10^7^

^a^Traces indicate absence of growth

**Fig. 1:**
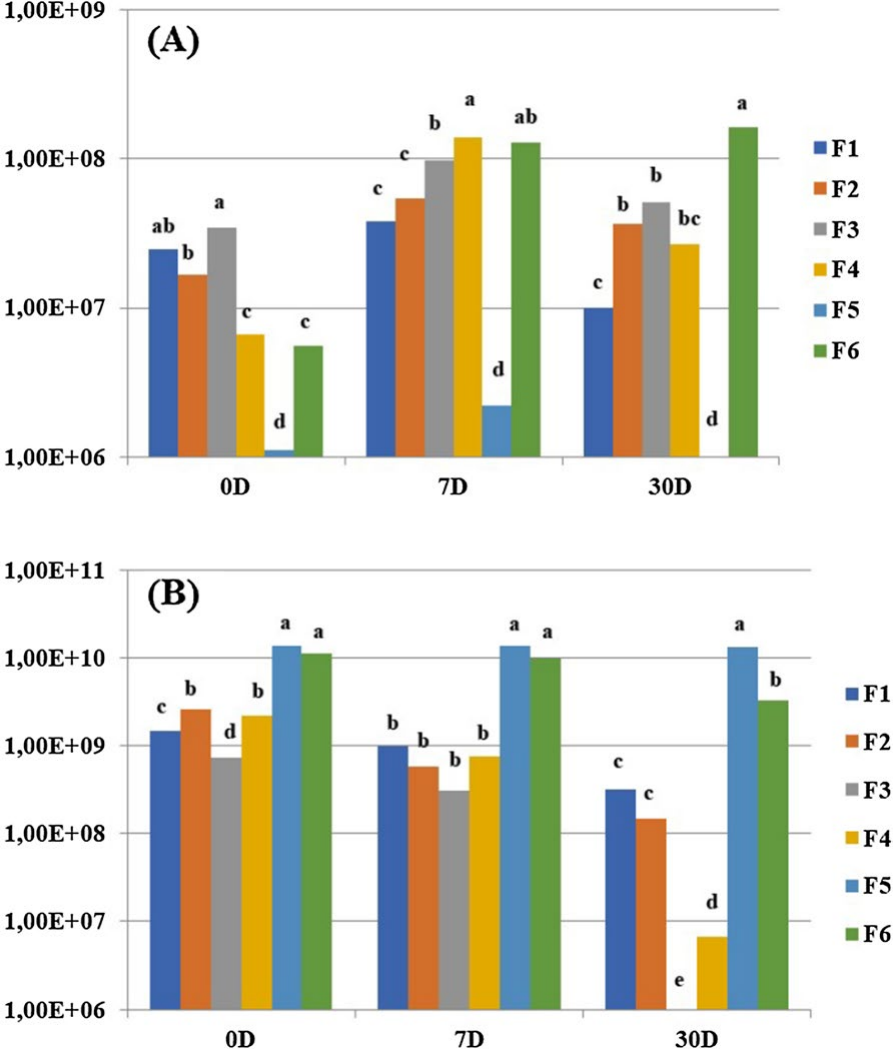
Colony forming units (CFU mL-1) of six formulation (F1 to F6) of co-inoculant containing (**A**) *Azospirillum* and (**B**) *Bradyrhizobium* at the end of the co-cultivation period (zero day, 0D), and after seven (7D) and 30 (30D) days of storage. Means of three replicates and diferente letter denote statistical difference between treatments within each genus (Tukey, *p* < 5%)

Based on the results obtained, formulations were evaluated (Table 1) to verify the growth capacity of *Bradyrhizobium* and *Azospirillum*, separately (data not shown). From the results obtained, six formulations were developed, which were evaluated for co-cultivation of *Bradyrhizobium* and *Azospirillum* for 30 days (Fig. 1). The results obtained indicated that it was possible to develop formulations that allow the co-cultivation of the two microorganisms. A formulation composed of malic acid, mannitol, yeast extract, CaCl_2_.2H_2_O, K_2_HPO, Mg.SO_4_.7H_2_O and NH_4_NO_3_ was then developed. Optionally, to allow higher cellular concentration, a micronutrient solution containing CuSO_4_.5H_2_O, ZnSO_4_.7H_2_O, H_3_BO_3,_ MnSO_4_.H_2_O e NaMoO_4_.2H_2_O can be added. The concentrations of each component in the formulation are subject to industrial registration.

The growth curves of *Bradyrhizobium* and *Azospirillum* differ considerably, with slow growth of the first and fast growth of the second. Therefore, in order to find a balance, it is necessary to adjust the inoculation time. In the test performed with different times of inoculation of *Azospirillum* in the formulation developed, the best results were obtained when *Azospirillum* was inoculated on the third day of growth of *Bradyrhizobium* (Table 4).

**Table 4:** Effect of different times of inoculation of *Azospirillum* on the growth (log of CFU) of *Bradyrhizobium* and *Azospirillum* co-cultivated in the same culture medium

Days of growth	*Bradyrhizobium*				*Azospirillum*			
	Single	Composite inoculant			Single	Composite inoculant		
	Control	Time of inoculation^a^			Control	Time of inoculation ^a^		
		T1	T2	T3		T1	T2	T3
2	9.34 b				8.00			
3	9.69 ab	9.16 c			8.11	8.00 a		
4	9.69 ab	9.19 bc	9.01 c		8.23	7.99 a	8.11 a	
5	10.14 a	9.13 c	9.67 ab	9.17 c	8.47	7.82 a	8.33 a	8.15 a

^a^Inoculation of *Azospirillum* after 2 (T1), 3 (T2) and 4 (T3) days of growth of *Bradyrhizobium*

### Field trials to evaluate agronomic efficiency

#### Characterization of the areas

The sites where the five experiments were performed represent distinct edaphoclimatic conditions of soybean-producing areas in Brazil. Regarding the main characteristics of the soils, the granulometry varied from highly clayey soils, in Londrina, with 75.2% clay in the 0–20 cm layer, to highly sandy soils, in Paranavaí, with 90.6% sand in the 0–20 cm layer (Additional file 1: Table S3). In general, Ca levels were low, but P levels ranged from low (Lutécia) to high (Londrina); Mn contents were high in all soils, except in Ponta Grossa (Additional file 1: Table S2).

Two areas were cropped for the first time and had never received inoculants before, and therefore showed low population of rhizobia compatible with soybean (Paranavaí and Lutécia), while the other areas had been previously cultivated and inoculated, harboring high populations, above 1.47 × 10^4^ rhizobia g^−1^ of soil (Additional file 1: Table S2).

All areas had high populations of diazotrophic bacteria, estimated to be at least 1.10 × 10^3^ bacteria g^−1^ soil (Additional file 1: Table S3).

### Field trials performed in areas without history of inoculation

In Lutécia (SP), in the evaluation carried out at the V5 stage, there was no nodulation in the non-inoculated treatments without (T1) and with (T2) N-fertilizer (Table 5). The composite inoculant developed in this study (T6) resulted in number of nodules comparable to those provided by the peat inoculant (T3) and by the commercial co-inoculation, represented by the separate supply of *Bradyrhizobium* spp. and *A. brasilense* (T5) and higher than the commercial liquid inoculant containing only *Bradyrhizobium* spp. (T4). Nodule dry weight of the T6 treatment was also comparable to T5, although lower than T3. Still at V5, the best performance of shoot dry weight and total N accumulated in shoots was verified in the treatment receiving N-fertilizer. At physiological maturity, the highest yields obtained in Lutécia were found with the peat inoculant (T3), commercial co-inoculation (T5), and the composite inoculant developed in this study (T6). Grain yield of the composite inoculant developed in this study exceeded the treatment receiving N-fertilizer by 26%. The two co-inoculants were the treatments that resulted in a greater mass of 100 grains. All inoculated treatments resulted in higher content and total N accumulated in the grains, statistically superior to the non-inoculated treatments receiving (T2) or not (T1) N-fertilizer (Table 5).

**Table 5:** Parameters evaluated in soybean cultivar BRS 1010IPRO with different treatments of inoculation with *Bradyrhizobium* and co-inoculation with *Bradyrhizobium* and *Azospirillum* in field trials performed in first-year cropping areas, without naturalized population of compatible soybean bradyrhizobia, in Lutécia-SP e Paranavaí-PR, Brazil

Treatment^a^	Stage V5					Physiological maturity			
	Nodulation		Shoot dry weight	N in shoot		N in grain		Grain yield	Mass of 100 grains
	n^o^ plant^−1^	mg plant^−1^	g plant^−1^	Content g kg^−1^	Total mg plant^−1^	Content g kg^−1^	Total kg ha^−1^	kg ha^−1^	g
Lutécia (SP)									
T1—Control-N	0.0 b^b^	0.0 c	6.71 b	21.0^n.s.b^	140.9 b	38.3 c	61 c	1601 c	14.4 d
T2—Control + 200 kg N	0.0 b	0.0 c	9.53 a	23.9	227.7 a	43.8 b	142 b	3244 b	15.7 c
T3—Peat Brady	12.5 a	54.7 a	4.73 b	25.3	119.7 b	52.9 a	215 a	4059 a	17.2 b
T4—Liquid Brady	4.9 b	30.5 b	4.70 b	22.0	103.4 b	51.6 a	181 a	3500 b	15.5 c
T5—Single Brady + Single Azo (trademark)	10.6 a	30.3 b	5.71 b	21.0	119.9 b	51.7 a	211 a	4086 a	18.0 a
T6—Composite inoculant Brady + Azo	10.5 a	33.8 b	6.02 b	25.8	155.3 b	50.7 a	208 a	4100 a	17.7 ab
*p* value	0.001	0.001	0.019	0.10	0.009	0.001	0.001	0.001	0.001
Paranavaí (PR)									
T1—Control—N	0.1 b	0.5 b	3.00 b	17.1 b	51.3 b	50.0 b	110 b	2202 d	12.6 a
T2—Control + 200 kg N	0.1 b	0.8 b	5.34 a	17.8 b	95.0 a	41.7 c	107 b	2574 cd	12.8 a
T3—Peat Brady	13.2 a	101.0 a	3.62 b	22.1 a	80.0 ab	54.0 a	157a	2904 bc	11.8 b
T4—Liquid Brady	8.5 a	74.2 a	3.60 b	21.0 a	75.6 ab	51.0 ab	143 a	2800 bc	11.5 b
T5—Single Brady + Single Azo (trademark)	10.7 a	76.2 a	4.32 ab	20.3 a	87.7 ab	52.9 a	166 a	3140 ab	12.5 a
T6—Composite inoculant Brady + Azo	8.6 a	77.6 a	3.92 ab	21.2 a	83.1 ab	52.0 a	181 a	3491 a	12.4 a
*p* value	0.001	0.001	0.038	0.007	0.097	0.001	0.001	0.001	0.003

^a^T3 e T4, peat and liquid inoculant with *Bradyrhizobium* spp. strains SEMIA 5079 + SEMIA 5080, applied to supply 1.2 × 10^6^ cells seed^−1^; T5, T4 + liquid inoculant with *A. brasilense* strains Ab-V5 + Ab-V6 applied to supply 1.2 × 10^5^ cells seed^−1^; T6, composite inoculant developed in this study, with *B. diazoefficiens* strain SEMIA 5080 (1.2 × 10^6^ cells seed^−1^) and *A. brasilense* strain Ab-V6 (1.2 × 10^5^ cells seed^−1^)

^b^Data represent the means of six replicates and when followed by different letters, within each column, are statistically different (Duncan, *p* < 5% or 10%; n.s., statistically non-significant)

In the other area cropped with soybean for the first time, in Paranavaí (PR), as expected, nodulation was practically absent in the non-inoculated plants, with or without N-fertilizer, in the evaluation carried out at V5 (Table 5). All inoculated treatments resulted in increases in nodule number and dry weight. Still at V5, the two co-inoculated treatments (T5 and T6) did not differ statistically from the treatment receiving N-fertilizer (T2), with higher values of shoot dry weight. All inoculated treatments increased the N content in shoots in comparison to the two non-inoculated controls (T1 and T2). In relation to the total N accumulated in shoots, T1 only differed statistically from the non-inoculated treatment receiving N-fertilizer (T2). At physiological maturity, the highest yield was obtained with the composite inoculant developed in this study, which did not differ statistically from the commercial co-inoculation with the two species applied separately. Both treatments of co-inoculation, T5 and T6 resulted in statistical difference in relation to the non-inoculated controls, with and without N-fertilizer, with the composite inoculant developed in this study (T6) being superior to the treatment receiving N-fertilizer by 36%. The grain yield of the treatment receiving the composite inoculant differed statistically from the single inoculation with *Bradyrhizobium* in liquid or peat formulation. As in Lutécia, all inoculated treatments resulted in higher contents and total N accumulated in grains in comparison to the non-inoculated controls without (T1) and with (T2) N-fertilizer (Table 5).

### Field trials performed in previously inoculated areas

In Florestópolis (PR), with a naturalized population estimated at 2.15 × 10^5^ rhizobia g^−1^ of soil (Additional file 1: Table S3), plants nodulated well, even in the non-inoculated controls, with no statistical differences in V5 (Table 6). Also, at V5 stage the highest shoot dry weight and total N accumulated in shoots were found in the non-inoculated treatments receiving N-fertilizer (T2), in the commercial liquid inoculant with *Bradyrhizobium* (T4) and in the composite inoculant developed in this study (T6). The composite inoculant resulted in the highest grain yield, statistically similar to the commercial co-inoculation with the isolated microorganisms. The composite inoculant developed in this study (T6) increased grain yield by 24% in comparison to the non-inoculated treatment (T1). All inoculated treatments resulted in increases in the N content in the grains and, in the case of the total N accumulated in the grains, they were statistically similar to the treatment receiving N-fertilizer (Table 6).

**Table 6:** Parameters evaluated in soybean cultivar BRS 1010IPRO with different treatments of inoculation with *Bradyrhizobium* and co-inoculation with *Bradyrhizobium* and *Azospirillum* in field trials performed in areas traditionally cropped with soybean, showing naturalized population of soybean bradyrhizobia, in Florestópolis-PR, Londrina-PR and Ponta-Grossa-PR, Brazil

Treatment^a^	Stage V5					Physiological maturity			
	Nodulation		Shoot dry weight	N in shoot		N in grains		Grain yield	Mass of 100 grains
	n^o^ plant^−1^	mg plant^−1^	g plant^-1^	Content g kg^−1^	Total mg plant^−1^	Content g kg^−1^	Total kg ha^−1^	kg ha^−1^	g
Florestópolis (PR)									
T1—Control-N	26.2^n.s.^^b^	64.3^n.s^	1.02 b^b^	29.6 a	30.2 ab	45.4 c	124 b	2722 b	15.47^n.s^
T2—Control + 200 kg N	25.3	65.1	1.61 a	29.7 a	47.8 a	46.6 b	147 ab	3158 ab	15.90
T3—Peat Brady	26.2	55.8	0.93 b	28.5 ab	26.5 b	48.3 ab	137 ab	2833 ab	15.33
T4—Liquid Brady	25.4	66.0	1.28 ab	26.9 c	34.4 ab	48.0 ab	131 ab	2725 b	15.50
T5—Single Brady + Single Azo (trademark)	26.8	62.9	0.98 b	26.7 c	26.2 b	49.8 a	165 a	3321 ab	15.60
T6—Composite inoculant Brady + Azo	29.4	71.9	1.22 ab	26.2 c	31.9 ab	49.0 a	165 a	3368 a	15.85
*p* value	0.10	0.10	0.095	0.02	0.090	0.001	0.095	0.090	0.10
Londrina (PR)									
T1—Control—N	56.9 a	207 a	9.32 ^n.s^	27.4 ^n.s^	255 ^n.s^	44.9 ^n.s^	184 ^n.s^	4101 ^n.s^	17.3 ^n.s^
T2—Control + 200 kg N	27.8 b	85 b	9.91	29.8	295	45.7	186	4067	17.7
T3—Peat Brady	54.8 a	215 a	8.66	31.9	276	45.3	187	4137	17.3
T4—Liquid Brady	55.0 a	210 a	8.50	31.8	270	45.1	184	4080	17.1
T5—Single Brady + Single Azo (trademark)	55.6 a	215 a	9.20	32.4	298	44.5	186	4193	17.0
T6—Composite inoculant Brady + Azo	46.3 a	188 a	8.56	31.0	265	44.8	184	4112	17.2
T7—T6 applied in-furrow	48.2 a	177 a	8.63	30.9	267	44.0	187	4260	17.1
*p* value	0.007	0.001	0.10	0.10	0.10	0.10	0.10	0.10	0.10
Ponta Grossa (PR)									
T1—Control-N	20.4 b	65.0 b	4.55 b	38.4 b	174 c	44.1 b	176 b	4001 b	16.6^n.s^
T2—Control + 200 kg de N	20.8 b	32.9 c	6.75 a	42.3 ab	286 a	43.7 b	177 b	4048 b	16.7
T3—Peat Brady	21.0 b	67.9 b	4.58 b	38.8 b	177 c	45.8 ab	184 b	4025 b	16.0
T4—Liquid Brady	20.2 b	66.0 b	4.33 b	38.9 b	168 c	46.0 ab	185 b	4020 b	16.1
T5—Single Brady + Single Azo (trademark)	28.9 ab	85.0 a	5.40 ab	42.0 ab	218 b	48.9 a	212 a	4335 ab	16.5
T6—Composite inoculant Brady + Azo	26.3 ab	85.8 a	5.44 ab	45.7 a	249 ab	48.7 a	222 a	4566 a	16.9
T7—T6 applied in-furrow	31.4 a	94.8 a	5.42 ab	46.6 a	252 ab	48.0 a	221 a	4528 a	16.7
*p* value	0.092	0.003	0.04	0.090	0.02	0.02	0.095	0.087	0.10

^a^T3 e T4, peat and liquid inoculant with *Bradyrhizobium* spp. strains SEMIA 5079 + SEMIA 5080, applied to supply 1.2 × 10^6^ cells seed^−1^; T5, T4 + liquid inoculant with *A. brasilense* strains Ab-V5 + Ab-V6 applied to supply 1.2 × 10^5^ cells seed^−1^; T6, composite inoculant developed in this study, with *B. diazoefficiens* strain SEMIA 5080 (1.2 × 10^6^ cells seed^−1^) and *A. brasilense* strain Ab-V6 (1.2 × 10^5^ cells seed^−1^); T7, treatment T6 applied in-furrow, 3 doses

^b^Data represent the means of six replicates and when followed by different letters, within each column, are statistically different (Duncan, *p* < 5% or 10%; n.s., statistically non-significant

In Londrina, the population was estimated at 1.47 × 10^3^ rhizobia g^−1^ of soil (Additional file 1: Table S3). No statistical differences were detected between treatments with or without inoculation in any of the parameters evaluated at V5, except for inhibition of nodulation by the application of N-fertilizer (T2) (Table 6). There were also no differences between treatments in grain yield and other parameters evaluated at physiological maturity. In Londrina, an extra treatment was performed with the in-furrow application of three doses of the composite inoculant developed in this study, but there was also no statistical difference between this treatment and the others. The results indicate that the naturalized population of *Bradyrhizobium* in the soil was able to provide the N necessary for the development of the plants, which is confirmed by the finding that there was no benefit from the application of N-fertilizer (Table 6).

In Ponta Grossa, the treatment with the composite inoculant developed applied in-furrow (T7) was also added and the three treatments with co-inoculation (T5, T6 and T7) resulted in the highest values of nodule number and dry weight at V5 (Table 5). Regarding nodule dry weight, there was inhibition by the application of N-fertilizer. In the same harvest, the two treatments with the composite inoculant developed in this study, T6 and T7, showed the best performance of N content and total N accumulated in shoots, not differing statistically from the N-fertilizer. At the physiological maturity, the highest yields were obtained with the composite inoculant developed in this study, applied on seeds (T6) or in-furrow (T7), not differing statistically from the commercial co-inoculation with the two separate bacteria (T5) and from the non-inoculated treatment with N-fertilizer (T2). The composite inoculant developed in this study resulted in statistically significant increase in grain yield, in relation to the non-inoculated control (T1), of 565 kg ha^−1^ and 527 kg ha^−1^ when applied on seeds (T6) or in-furrow (T7), respectively. The developed composite inoculant also statistically increased grain yield in relation to the non-inoculated control receiving N-fertilizer (T2), the peat inoculant (T3) and the liquid inoculant (T4) containing only *Bradyrhizobium* spp. It should also be noted the higher values of total N accumulated in the grains obtained in all co-inoculated treatments and the increase in the N content in the grains in all inoculated treatments (Table 5).

**Fig. 2:**
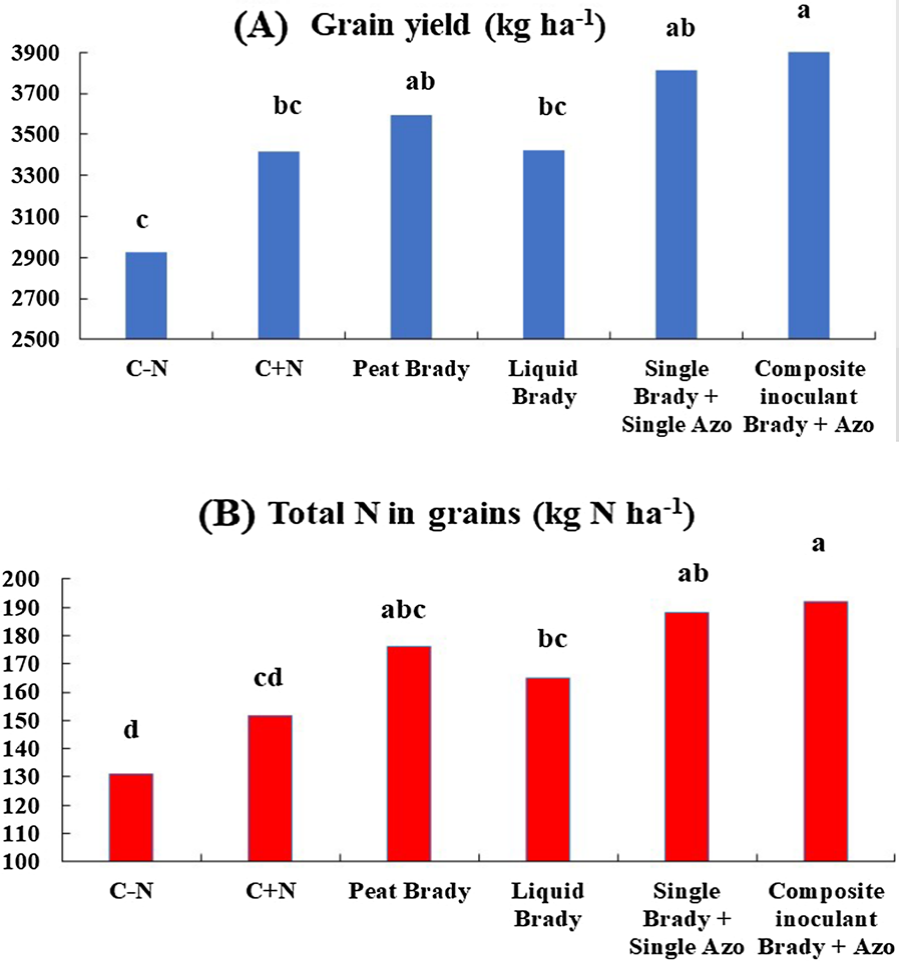
**A** Grain yield and (**B**) total N accumulated in soybean grains resulting from single inoculation with peat and liquid inoculant of *Bradyrhizobium*, in co-inoculation with single *Bradyrhizobium* and single *Azospirillum* and with the co-inoculant carrying both *Bradyrhizobium* and *Azospirillum*. Data represent the means of five field experiments, each with six replicates and when followed by the same letter are not statistically diferente (Duncan, *p* < 5%)

In a joint analysis of the five trials, considering the average grain yield, the composite inoculant developed in this study was statistically superior to the non-inoculated controls without and with N-fertilizer and to the commercial liquid inoculant containing only *Bradyrhizobium*, and equal to the commercial co-inoculation with the bacteria used separately (Fig. 2). Similar results were obtained in the joint analysis of the total N accumulated in grains, with the composite inoculant resulting in statistically significant increases of 27, 40 and 61 kg of N in grains ha^−1^ in comparison to the liquid inoculant carrying only *Bradyrhizobium* spp. and to the non-inoculated controls with and without N-fertilizer, respectively (Fig. 2).

## Discussion

Although sucrose, mannitol, and glycerol were the C sources that yielded higher cell concentrations, growth of *Bradyrhizobium* in sucrose as a primary source of C was not expected, since the genus reportedly lacks the invertase enzyme, which is necessary for cells to metabolize sucrose, rhamnose and trehalose (Vincent 1977; Martinez-Drets and Arias 1974). For that reason, sucrose has been used by some inoculant industries as a cell protector of soybean *Bradyrhizobium*, and not as a C source.

One hypothesis to explain the good growth observed in our experiments could be the possible hydrolysis of sucrose by hydrogen ions (H^+^) derived from the dissociation of the molecule at high temperatures, in the process of autoclaving the culture medium. Another hypothesis is that, even with low dissociation, the remaining sucrose would be able to provide protection to the cells, favoring survival and contributing to greater cellular concentration. It is also important to search in the genome of the two strains of *Bradyrhizobium* used in this study (Hungria et al. 2018) for possible alternative routes of assimilation of sucrose. Mannitol is broadly known as an appropriate source of C for *Bradyrhizobium* and the same occurs with glycerol, which is also the source of preference in several inoculant industries (Vincent 1970, 1977; Lopreto et al. 1972; Balatti 1992; Balatti and Freire 1996; Hungria et al. 2016).

The growth of *A. brasilense* was maximized in a culture medium containing malic acid and maleic acid; in fact, the preferred use of malic acid by this species is broadly known (Döbereiner et al. 1995). However, the high cost of maleic acid makes it unattractive for utilization in the commercial production of inoculants. There was also good growth in the presence of mannitol, which is justified by the presence of the enzyme mannitol dehydrogenase (Westby et al. 1983). According to Döbereiner and Pedrosa (1987), and Hartmann and Baldani (2006), sucrose would not be a good source of C for *A. brasilense*; however, in our study, good growth was verified with this source. As for *Bradyrhizobium*, it is necessary to investigate whether the growth of *A. brasilense* is due to partial hydrolysis of sucrose, or to metabolic pathways for the use of sucrose.

Brazil plays an increasingly prominent role in the international agricultural scenario due to the use of inoculants in soybean crops, with high economic and environmental returns for farmers and for the country (Hungria et al. 2006, 2007; Hungria and Mendes 2015; Hungria and Nogueira 2019). Increasing success has been obtained after the deployment of the technology of co-inoculation with *Bradyrhizobium* spp. and *A. brasilense* in 2013, with doubled grain yield benefits culminating in the commercial launch of a technological package containing the two bacteria separately (Hungria et al. 2013b, 2015; Barbosa et al. 2021; Santos et al. 2021). In a recent a meta-analysis of 48 publications covering field trials at 38 different locations in Brazil, Barbosa et al. (2021) detected statistically significant increases in grain yield and other plant growth parameters due to the co-inoculation. In addition, the main published studies in Brazil were recently compiled by Santos et al. (2021), and as an example, the studies of Ferri et al. (2017) and Galindo et al. (2018) indicated increases in grain yield due to the co-inoculation of 20.3 and 11.2%, respectively, compared to the inoculation exclusively with *Bradyrhizobium*. In the co-inoculation, although there may be a contribution from *A. brasilense* via biological nitrogen fixation, the benefits provided by strains Ab-V5 and Ab-V6 have been mainly attributed to the production of phytohormones, resulting in expressive increases in several root parameters (Fukami et al. 2018b; Rondina et al. 2020). Root growth increases also favor the uptake of fertilizers (Galindo et al. 2021). There are also reports of induction of plant tolerance to abiotic stresses (Fukami et al. 2017; 2018a).

In addition to scientific evidence, the success of a new agricultural technology depends on large-scale proof of benefits to farmers. In this context, field extension efforts have been applied for three growing seasons in the state of Paraná, Brazil, with the establishment of reference units (RU) and field days. In the first year, 2017/2018, 37 RU were installed in 23 municipalities, assisting 665 farmers. Co-inoculation with *Bradyrhizobium* spp. and *A. brasilense* resulted in average increase in grain yield of 228 kg ha^−1^ with profit of R$ 263.4 ha^−1^ (~ U$ 70 in 05/18) in relation to the single inoculation with *Bradyrhizobium* (Nogueira et al. 2018). In the following season (2018/2019), in 61 RU located in 46 municipalities assisting 925 producers, co-inoculation resulted in average yield increase of 259 kg ha^−1^, and net profit of R$ 296.00 ha^−1^ (~ U$ 76 in 05/19) (Prando et al. 2019) and, in the third season (2019/2020), these numbers were of 63 RU, in 54 municipalities, assisting 636 farmers, average gain of 266 kg ha^−1^ and profit of R$ 348.23 ha^−1^ (~ U$ 64 on 05/20) (Prando et al. 2020). The large-scale confirmation of benefits explains the widespread adoption of co-inoculation in the country in a short time, estimated at 15% of the entire cultivated area in 2018/2019, increasing to 25%, or almost 9 million ha in 2019/2020 (Santos et al. 2021).

The success of co-inoculation in Brazil finds limitations in the use of microorganisms packaged separately, with a great demand, mainly by medium and small farmers, for composite inoculants. This is just one example, as the demand for composite inoculants, containing microorganisms with different metabolic functions, grows internationally (Santos et al. 2019). However, the development of inoculants containing microorganisms with different metabolic needs and growth rates is challenging. In this study, the feasibility of combining microorganisms with different metabolic needs and growth rates was demonstrated, through the development of a proper formulation composed by viable C sources and definition of times of inoculation. In our study, the preferred C sources for *Bradyrhizobium* were glycerol and mannitol, and for *A. brasilense* malic acid. In addition, as the growth rates were different, the best results were obtained with the inoculation of the fast-growing *A. brasilense* on the third day of growth of *Bradyrhizobium*.

In five field trials, the developed composite inoculant showed performance similar to that of co-inoculation with the two microorganisms provided separately, resulting in average increase in grain yield of 502 kg ha^−1^, or 14.7% in relation to the liquid inoculant containing only *Bradyrhizobium* (Fig. 2).

It should also be noted that there is great concern in the agribusiness sector about the low levels of protein in soybean grains and there are indications that the N from the BNF is more easily translocated to grains than the N-fertilizer (Hungria and Neves 1987; Kaschuk et al. 2010; Hungria et al. 2020). This was confirmed in the field trials performed in our study, with an average increment of 16.4% in the total N accumulated in the grains in response to inoculation and co-inoculation (Fig. 2).

The development of composite microbial inoculants and extension activities with the farmers showing the benefits of microbial inoculants should be encouraged globally, in view of several reports, including this one, showing agronomic, economic, and environmental benefits by the replacement of chemical fertilizers.

## Supplementary Information

**Additional file 1: Table S1.** Geographic coordinates, climate conditions, properties and classification of the soil in each site where the field experiments were performed. **Table S2.** Soil chemical properties in the 0–20 e 20–40 cm layers in the experimental sites before sowing. **Table S3.** Soil chemical characterization and granulometry in the 0–20 e 20–40 cm layers and population of rhizobia symbionts of soybean and of diazotrophic bacteria in the 0–10 cm layer in the sites of the experiments before sowing. **Table S4.** Agronomic information about the field experiments.

## Data Availability

All datasets generated or analyzed during this study are included in the manuscript, and complementary dataset will be available upon request to the corresponding author.
