# Supplementary material for: Combining microorganisms in inoculants is agronomically important but industrially challenging: case study of a composite inoculant containing Bradyrhizobium and Azospirillum for the soybean crop
Source: AMB Express. 2021 May 22;11:71. doi: 10.1186/s13568-021-01230-8 (PMC8141083; doi:10.1186/s13568-021-01230-8)
Supplement: Supplementary file 1 — Additional file 1: Table S1. Geographic coordinates, climate conditions, properties and classification of the soil in each site where the field experiments were performed. Table S2. Soil chemical properties in the 0–20 e 20–40 cm layers in the experimental sites before sowing. Table S3. Soil chemical characterization and granulometry in the 0–20 e 20–40 cm layers and population of rhizobia symbionts of soybean and of diazotrophic bacteria in the 0–10 cm layer in the sites of the experiments before sowing. Table S4. Agronomic information about the field experiments. [file 13568_2021_1230_MOESM1_ESM.docx]

**Table S1 Geographic coordinates, climate conditions, properties and classification of the soil in each site where the field experiments were performed.**

| **Site** | **Coordenates^a^** | **Altitude**  (m) | **Climatic classification^b^** | **Average minimum temperature** (ºC) | **Average maximum temperature**  (ºC) | **Annual rainfall**  (mm) | **Soil properties** | **North-American soil classification** |
| --- | --- | --- | --- | --- | --- | --- | --- | --- |
| Lutécia (SP) | 22° 11' 45,58" S,  50° 25' 27,82" O | 470 | *Cfa* | 18.2 | 21.2 | 1,143 | sandy | Typic Acrudox^c^ |
| Paranavaí (PR) | 22° 57' 16,82" S  52° 28' 4,31" O | 400 | *Cfa* | 19.1 | 29.4 | 1,457 | clayey | Typic Acrudox^c^ |
| Florestópolis (PR) | 22° 50' 21,89" S  51° 27' 25,08" O | 494 | *Cfa* | 18.5 | 28.6 | 1,440 | sandy | Typic Acrudox^c^ |
| Londrina (PR) | 23° 11' 2,75" S  51° 10' 30,29" O | 595 | *Cfa* | 13.3 | 28.5 | 1,651 | clayey | Rhodic Eutrudox^d^ |
| Ponta Grossa (PR) | 25° 8' 52,01" S  50° 4' 43,93" O | 880 | *Cfb* | 8.4 | 25.9 | 1,507 | sandy | Typic Acrudox^c^ |

^a^ Latitude, longitude

^b^ After the classification of Köppen-Geiger; *Cfa*, humid subtropical; *Cfb,* temperate; *Aw*, tropical with dry season in the winter

^c^ Brazilian classification, Latossolo Vermelho Distrófico

^d^ Brazilian classification*,* Nitossolo Vermelho Eutroférrico

**Table S2 Soil chemical properties in the 0-20 e 20-40 cm layers in the experimental sites before sowing.**

| **Site** | **Depth** | **pH** | **pH** | **Potential acidity** | **Acidity** | **Ca^+2^** | **Mg^2+^** | **K^+^** | **Na^+^** | **P**  **Mehlich1** | **P Mehlich3** | **Cu**^2+^ | **Fe**^2+^ | **Mn**^2+^ | **Zn**^2+^ |
| --- | --- | --- | --- | --- | --- | --- | --- | --- | --- | --- | --- | --- | --- | --- | --- |
|  |  | (CaCl_2_) | (H_2_O) | (H + Al) | (H^+^) |  |  |  |  |  |  |  |  |  |  |
|  |  |  |  | -------------------- cm_c_ dm^-3^ --------------------- | | | | | | ---- mg dm^-3^ --- | | ------------- mgdm^-3^ -------------- | | | |
| Lutécia | 00-20 | 4.71 | 5.41 | 2.78 | 2.78 | 0.85 | 0.41 | 0.07 | 0.01 | 5.15 | 7.12 | 0.88 | 130.29 | 69.44 | 1.01 |
|  | 20-40 | 4.19 | 4.95 | 2.99 | 2.99 | 0.79 | 0.48 | 0.04 | 0.01 | 4.39 | 5.60 | 0.91 | 153.52 | 61.59 | 1.31 |
| Paranavaí | 00-20 | 5.17 | 5.80 | 2.38 | 2.38 | 1.00 | 0.57 | 0.17 | 0.01 | 19.17 | 26.96 | 1.10 | 124.20 | 165.40 | 5.19 |
|  | 20-40 | 4.81 | 5.49 | 2.52 | 2.52 | 0.73 | 0.43 | 0.10 | 0.02 | 4.04 | 7.78 | 1.09 | 129.05 | 158.37 | 8.39 |
| Florestópolis | 00-20 | 5.04 | 5.69 | 2.50 | 2.50 | 1.16 | 0.59 | 0.14 | 0.01 | 57.85 | 66.07 | 1.70 | 153.58 | 152.16 | 2.23 |
|  | 20-40 | 3.84 | 4.65 | 3.58 | 3.58 | 0.42 | 0.14 | 0.11 | 0.01 | 12.32 | 13.77 | 2.32 | 130.75 | 151.21 | 1.23 |
| Londrina | 00-20 | 5.20 | 5.83 | 5.31 | 5.31 | 3.75 | 2.00 | 0.84 | 0.01 | 22.53 | 13.84 | 12.93 | 101.30 | 445.05 | 2.04 |
|  | 20-40 | 5.07 | 5.72 | 5.35 | 5.35 | 3.22 | 1.73 | 0.37 | 0.01 | 11.40 | 6.49 | 11.19 | 98.61 | 316.08 | 1.28 |
| Ponta | 00-20 | 4.64 | 5.34 | 6.35 | 6.35 | 2.96 | 1.02 | 0.36 | 0.01 | 20.75 | 16.98 | 1.38 | 127.50 | 17.29 | 1.13 |
| Grossa | 20-40 | 4.40 | 5.14 | 6.74 | 6.74 | 1.64 | 0.62 | 0.27 | 0.01 | 2.97 | 2.33 | 1.08 | 110.06 | 12.43 | 0.50 |

**Table S3 Soil chemical characterization and granulometry in the 0-20 e 20-40 cm layers and population of rhizobia symbionts of soybean and of diazotrophic bacteria in the 0-10 cm layer in the sites of the experiments before sowing.**

| Site | Depth | CEC (cmol_c_ dm^-3^)^a^  -------------------------------- | | Bases saturation | Carbon | Granulometry (%)  ------------------------------------------- | | | Population of rhizobia | Population of diazotrophic^b^ |  |
| --- | --- | --- | --- | --- | --- | --- | --- | --- | --- | --- | --- |
|  |  | pH 7,0 | efetiva | V (%) | (g dm^-3^) | Clay | Silt | Sand | (rhizobia g^-1^) | (bacteria g^-1^) | |
| Lutécia | 00-20 (10 ^a^) | 4.12 | 1.34 | 32 | 4.14 | 11.9 | 1.1 | 86.9 | 7.36 × 10^1^ | 9.50 × 10^3^ | |
|  | 20-40 | 4.31 | 1.32 | 30 | 4.07 | 9.95 | 1.0 | 89.0 | n.e.^c^ | n.e. | |
| Paranavaí | 00-20 (10) | 4.13 | 1.75 | 42 | 4.50 | 6.5 | 2.85 | 90.6 | zero | 2.50 × 10^3^ | |
|  | 20-40 | 3.80 | 1.28 | 33 | 2.36 | 8.5 | 2.1 | 89.4 | n.e. | n.e. | |
| Florestópolis | 00-20 (10) | 4.40 | 1.90 | 43 | 2.89 | 8.5 | 2.35 | 89.1 | 2.15 × 10^5^ | 1.10 × 10^3^ | |
|  | 20-40 | 4.26 | 0.68 | 16 | 2.82 | 16.9 | 1.25 | 81.8 | n.e. | n.e. | |
| Londrina | 00-20 (10) | 11.9 | 6.60 | 55 | 19.1 | 75.2 | 18.5 | 6.25 | 1.47× 10^4^ | 1.47 × 10³ | |
|  | 20-40 | 10.6 | 5.33 | 50 | 13.9 | 80.1 | 14.1 | 5.8 | n.e. | n.e. | |
| Ponta Grossa | 00-20 (10) | 10.7 | 4.35 | 41 | 23.4 | 49.3 | 6.45 | 44.2 | 1.10× 10^6^ | 9.50 × 10^3^ | |
|  | 20-40 | 9.28 | 2.54 | 27 | 19.8 | 52.2 | 6.65 | 41.1 | n.e. | n.e. | |

^a^ CEC, cation exchange capacity

^b^ Evaluation free-living diazotrophic bacteria

^c^ Non-evaluated (n.e.)

**Table S4 Agronomic information about the field experiments**

| **Site** | **Cultivar** | **Sowing date** | **Plant density**  (plants ha^-1^) | **Vegetative harvest** | **Stage of vegetative harvest** | **Grain harvest** | **Total area of each replicate** | **Area harvested for grain yield** |
| --- | --- | --- | --- | --- | --- | --- | --- | --- |
| Florestópolis | BRS 1010IPRO | 18/10/2017 | 333,333 | 29/11/2017 | 42 DAS^a^, 35 DAE^a^, V5^b^ | 27/02/2018 | 25.2 m^2^ | 7.425 m^2^ |
| Paranavaí | BRS 1010IPRO | 01/11/2017 | 333,333 | 14/12/2017 | 43 DAS, 35 DAE, V5 | 05/03/2018 | 24 m^2^ | 6.75 m^2^ |
| Lutécia | BRS 1010IPRO | 07/11/2017 | 333,333 | 20/12/2017 | 42 DAS, 35 DAE, | 08/03/2018 | 25.2 m^2^ | 6.75 m^2^ |
| Londrina | BRS 1010IPRO | 15/11/2017 | 240,000 | 27/12/2017 | 42 DAS, 35 DAE, V5 | 24/03/2018 | 24 m^2^ | 8.0 m^2^ |
| Ponta Grossa | BRS 1010IPRO | 22/11/2017 | 240,000 | 10/01/2018 | 49 DAS, 36 DAE, V5 | 10/04/2018 | 24 m^2^ | 8.0 m^2^ |

^a^ Days after sowing (DAS), days after emergence (DAE)

^b^ Stage V5 of soybean growth (Fehr and Caviness, 1977)
